# EhVps35, a retromer component, is a key factor in secretion, motility, and tissue invasion by *Entamoeba histolytica*

**DOI:** 10.3389/fcimb.2024.1467440

**Published:** 2024-09-27

**Authors:** Joselin Díaz-Valdez, Rosario Javier-Reyna, Ausencio Galindo, Lizbeth Salazar-Villatoro, Sarita Montaño, Esther Orozco

**Affiliations:** ^1^ Departamento de Infectómica y Patogénesis Molecular, Centro de Investigación y de Estudios Avanzados del Instituto Politécnico Nacional (IPN), Ciudad de México, Mexico; ^2^ Laboratorio de Bioinformática y Simulación Molecular, Facultad de Ciencias Químico-Biológicas, Universidad Autónoma de Sinaloa, Sinaloa, Mexico

**Keywords:** vesicular trafficking, ESCRT machinery, retromer, *Entamoeba histolytica*, virulence mechanisms, EhVps35

## Abstract

In humans and *Drosophila melanogaster*, the functional convergence of the endosomal sorting complex required for transport (ESCRT) machinery that is in charge of selecting ubiquitinated proteins for sorting into multivesicular bodies, and the retromer, that is the complex responsible for protein recycling to the plasma membrane and Golgi apparatus. ESCRT and retromer complexes are codependent for protein sorting recycling, degradation, and secretion. In this article, we studied the EhVps35 C isoform (referred to as EhVps35), that is the central member of the *Entamoeba histolytica* retromer, and its relation with the ESCRT machinery during sorting and protein recycling events and their involvement virulence. Our findings revealed that EhVps35 interacts with at least 300 proteins that participate in multiple cellular processes. Laser confocal and transmission electronic microscopy images, as well as secretion assays, revealed that EhVps35 is secreted in vesicles together with EhVps23 and EhADH (both ESCRT machinery proteins). In addition, immunoprecipitation, immunofluorescence, and molecular docking assays revealed the relationship among EhVps35 and other ESCRT machinery proteins. Red blood cell stimulus increased EhVps35 secretion, and the knockdown of the *Ehvps35* gene in trophozoites reduced their capacity to migrate and invade tissues. This also impacts the cellular localization of ubiquitin, EhVps23 (ESCRT-I), and EhVps32 (ESCRT-III) proteins, strongly suggesting their functional relationship. Our results, taken together, give evidence that EhVps35 is a key factor in *E. histolytica* virulence mechanisms and that it, together with the ESCRT machinery components and other regulatory proteins, is involved in vesicle trafficking, secretion, migration, and cell proliferation.

## 1 Introduction

Two evolutionarily conserved cellular machineries, the endosomal sorting complex required for transport (ESCRT) and the retromer complex, mediate cargo sorting into the degradative and recycling pathways, respectively (Cullen and Steinberg, 2018). The ESCRT machinery is formed by the ESCRT-0, ESCRT-I, ESCRT-II, and ESCRT-III complexes, and accessory proteins (Leung et al., 2008), while the retromer is in general composed of five proteins that form two subcomplexes: the cargo selective complex shaped by the Vps26, Vps29, and Vps35 proteins and the SNX-BAR dimer (Vps5 and Vps17 in yeast) (Seaman, 2021). The retromer and ESCRT machinery participate together in cell division, endocytosis, secretion, migration, and regulated cell death, among many other cellular processes, and, in humans, alterations of these machineries produce diseases (Tomavo et al., 2013; Wang and Bellen, 2015; Gras et al., 2019; Vietri et al., 2019; Seaman, 2021; Tan et al., 2022; Yang et al., 2022). Recently, it has been reported that the functions of the ESCRT machinery and the retromer complex are mutually codependent, since the silencing of the TSG101 and CHMP3 proteins (Vps23 and Vps32, respectively in yeast), both components of the ESCRT machinery, inhibits the recycling of cargoes by the retromer (Dukes et al., 2011; Pannen et al., 2020); while, *vps35* gene silencing causes a negative regulation of degradation pathways, augments protein ubiquitination, and alters the exosome secretion (Williams et al., 2018; Liu et al., 2020; Filippone et al., 2021b, 2021a; Walsh et al., 2021; Tan et al., 2022) produced through the release of multivesicular bodies (MVBs) intraluminal vesicles, formed by the ESCRT machinery (Anand et al., 2019).

*Entamoeba histolytica*, the protozoan responsible for human amoebiasis, presents very active vesicular trafficking and membrane movement in the basal state, and they increase during phagocytosis and tissue invasion. The constant membrane remodeling in this parasite is crucial for cellular functions, and the ESCRT and the retromer complexes actively participate in these events (Arroyo and Orozco, 1987; García-Rivera et al., 1999; Nakada-Tsukui et al., 2005; Bañuelos et al., 2012; Avalos-Padilla et al., 2015, 2018; Ocádiz-Ruiz et al., 2016; Srivastava et al., 2017; Watanabe et al., 2020; Galindo et al., 2021, 2022; Díaz-Hernández et al., 2023; Díaz-Valdez et al., 2024). Our group has studied the participation of the ESCRT components in phagocytosis and other virulence processes in trophozoites. In addition, others have investigated the retromer components in *E. histolytica*, identifying the proteins EhVps35 (corresponding to the EhVps35C isoform reported in Díaz-Valdez et al., 2024), EhVps26, and EhVps29 (Nakada-Tsukui et al., 2005; Srivastava et al., 2017; Watanabe et al., 2020). Using two-hybrid screening, they demonstrated a direct interaction between these proteins. Furthermore, the EhVps26, EhVps29, and SNX1 proteins participate in phagocytosis (Nakada-Tsukui et al., 2005; Srivastava et al., 2017; Watanabe et al., 2020). We have recently published a study on the function of EhVps35 (EhVps35C isoform) in phagocytosis and EhADH and Gal/GalNac in lectin recycling (Díaz-Valdez et al., 2024), two virulence-involved proteins (Arroyo and Orozco, 1987; García-Rivera et al., 1999; Petri et al., 2002; Frederick and Petri, 2005; Bañuelos et al., 2012). Our findings also showed that EhVps35 is involved in cytoskeleton structuration because the knockdown of the *Ehvps35C* gene (*Ehvps35*-KD) caused cytoskeleton disruption and decreased phagocytosis (Díaz-Valdez et al., 2024). In addition, experimental evidence on the interaction of EhVps23 with EhVps35 (Galindo et al., 2022) and of EhADH with EhVps35 (Díaz-Valdez et al., 2024) strengthened the evidence of the relationship between the ESCRT and the retromer.

Here, we furthered the study of the ESCRT-retromer interaction using trophozoites in a basal state and after cellular stimulus with red blood cells (RBCs). Docking analysis and immunoprecipitation, using α-EhVps35 antibodies (specifically recognizes EhVps35C), substantiated that the EhTom1 (ESCRT-0), EhVps23 (ESCRT-I), and EhVps32 (ESCRT-III) proteins, and EhADH (an ESCRT accessory protein, belonging to the ALIX family, characterized by the Bro-1 domain) interact with EhVps35. Experiments using *Ehvps35*-KD trophozoites evidenced the codependence between the ESCRT and the retromer complexes because the gene silencing altered EhVps23 and EhVps32 cellular locations, affecting the formation and motility of MVBs and the capacity of trophozoites to produce hepatic abscesses. These functions revealed that EhVps35 may be a new target for anti-amoebiasis drug design.

## 2 Materials and methods

### 2.1 E. histolytica culture

*E. histolytica* trophozoites, strain HM1: IMSS, were axenically grown at 37°C in TYI‐S‐33 medium (Diamond et al., 1978) and harvested at the logarithmic growth phase. The culture flasks were then chilled at 4°C, and trophozoites were collected by centrifugation. All experiments reported here were performed at least three times in independent experiments, with two technical replicates per experiment.

### 2.2 Antibodies

The amino acid sequence of EhTom1 (access numbers: C4LXU1), described by Bañuelos et al. (2022) as a component of ESCRT, was used to design a specific peptide to generate mouse polyclonal antibodies against a specific EhTom1 peptide (N_244-_ EQIKTTLERHKKLTEK-C_259_). Male BALB/c mice (from an already-existing collection in the Unidad de Producción y Experimentación de Animales de Laboratorio-Centro de Investigación y de Estudios Avanzados del Instituto Politécnico Nacional (UPEAL-CINVESTAV) were immunized with 80 µg of the peptide resuspended in TiterMax Gold adjuvant (1:1) (Sigma Aldrich). Three more immunizations were then performed at 15-day intervals, followed by bleeding to obtain the α-EhTom1 antibody. Pre-immune serum was obtained before the immunizations.

The other primary antibodies used in this study were mouse α-EhVps35, which specifically recognizes the EhVps35C isoform (onwards the EhVps35 protein) (Díaz-Valdez et al., 2024), mouse monoclonal α-Ubiquitin (α-Ub) (Santacruz), rabbit α-EhADH (Galindo et al., 2022), mouse monoclonal α-human actin (kindly given by Dr. Manuel Hernandez, CINESTAV IPN), rabbit α-EhCP112 (García-Rivera et al., 1999), rat α-EhVps23 (Galindo et al., 2021), mouse α-EhVps32 (Avalos-Padilla et al., 2015), and rabbit α-EhVps36 (Díaz-Hernández et al., 2023). As secondary antibodies, we used HRP-labeled α-mouse IgG, α-rabbit IgG, and α-rat IgG (Zymed) for the Western blot assays; and Pacific Blue-labeled or Alexa Fluor 647-labeled α-mouse IgG and Cy5-labeled α-rat IgG (Life Technologies) for the immunofluorescence assays. For the immunoelectron microscopy experiments, we used α-mouse IgG conjugated with 20 nm or 30 nm gold particles, α-rat IgG conjugated with 10 nm gold particles, and α-rabbit IgG conjugated with 30 nm gold particles (TED Pella Inc).

### 2.3 Immunoprecipitation assays

Immunoprecipitation was carried out with 200 µl of protein-G-agarose (rProtein-G; Invitrogen) previously incubated for 2 h at 4°C with the α-EhVps35 antibody or preimmune serum (Díaz-Valdez et al., 2024). Trophozoite lysates were prepared in the presence of 10 mM Tris-HCl, 50 mM NaCl, and a protease inhibitors cocktail during freeze-thawing in liquid nitrogen and vortexing cycles. The lysates were then pre-cleared with 200 µl of rProtein-G (previously blocked with 2% BSA) and incubated for 2 h at 4°C under gentle stirring (Avalos-Padilla et al., 2015; Galindo et al., 2021). The previously cleared cell lysates were incubated overnight (ON) at 4°C with rProtein-G bound to α-EhVps35 antibody, and then the complex: rProtein-G/α-EhVps35/EhVps35-associated proteins were recovered by centrifugation. After washing with PBS, 60 µl of 4 x sample buffer (40% glycerol, 240 mM Tris-HCl, pH 6.8, 8% SDS, 0.04% bromophenol blue, and 5% of β-mercaptoethanol). Samples were boiled for 3 min and centrifuged again at 11,600 g for 2 min at 4°C. The supernatant (30 μl) was loaded into 10% SDS–PAGE and subjected to Western blot assays using the α-EhVps35, α-EhTom1, α-EhVps23, α-EhVps36, α-EhVps32, and α-EhADH antibodies. In addition, replicas of the same experiment were analyzed in the Proteomics Units of the LaNSE (National Laboratory of Experimental Services) of CINVESTAV to massively identify the proteins that bind to EhVps35.

### 2.4 Mass spectrometry analysis

In total, 30 μg of the immunoprecipitated proteins with the α-EhVps35 antibody were enzymatically digested according to the protocol reported by Ramírez-Flores et al. (2019). Afterward, the peptides were loaded into a Symmetry C18 Trap V/M precolumn (Waters); 180 μm × 20 mm, 100 Å pore size, 5 μm particle size, and desalted using mobile phase A (0.1% formic acid in H2O) and mobile phase B (0.1% formic acid in acetonitrile) under the following isocratic gradient: 99.9% mobile phase A and 0.1% of mobile phase B at a flow of 5 μl/min for 3 min. Then, the peptides were loaded and separated on an HSS T3 C18 column (Waters); 75 μm × 150 mm, 100 Å pore size, 1.8 μm particle size, using an UPLC ACQUITY M-Class (Waters) with the same mobile phases under the following gradient: 0 min 7% B, 121.49 min 40% B, 123.15 to 126.46 min 85% B, and 129 to 130 min 7% B, at a flow of 400 nL/min and at 45°C. The spectra data were acquired in a mass spectrometer, Synapt G2-Si (Waters), with electrospray ionization and ion mobility separation using a data-independent acquisition approach through the HDMSE mode (Waters). The generated raw files containing MS and MS/MS spectra were deconvoluted and compared using ProteinLynx Global Server (PLGS) v3.0.3 software (Li et al., 2009) against a reversed *E. histolytica* database (downloaded from Uniprot). Only the proteins with ≥ 95% reliability (Protein AutoCurate green) were reported here, according to the methodology described by Ramírez-Flores et al. (2019). Additionally, the identified proteins were classified according to their function, as described in the literature, and analyzed using the PANTHER server and the GeneOntology (GO) database (http://geneontology.org/). GO analysis was conducted using the top 10 enriched GO terms in the biological processes, molecular functions, and cellular component branches. All the adjusted and statistically significant P values of the terms were log-normalized negative 10 bases.

### 2.5 Phagocytosis assays

For the phagocytosis assays, trophozoites were incubated with RBCs (1:25) from an already-existing collection for 2 min at 37°C. The cell mixture was then washed with TYI-water (2:1) at 37°C to remove the adhered and non-ingested RBCs. Subsequently, the cells were incubated at 37°C for 28 min and the samples were processed for the immunofluorescence assays (García-Rivera et al., 1982).

### 2.6 Laser confocal microscopy assays

Trophozoites were grown on coverslips, fixed with 4% paraformaldehyde at 37°C for 1 h, permeabilized with 0.2% Triton X-100, and blocked with 10% fetal bovine serum in PBS. Preparations were incubated at 4°C ON with primary antibodies (1:50); and were incubated with the corresponding secondary antibodies for 30 min at 37°C: Pacific Blue-labeled α-mouse IgG for α-Ub, Cy5-labeled α-rat IgG for α-EhVps23 or Alexa Fluor 647-labeled α-mouse IgG for α-EhVps32 (1:100). We directly labeled the α-EhVps35 antibody with the FITC labeling fluorochrome kit (Molecular Probes-Thermo Fisher). All preparations were preserved using the Vectashield antifade reagent (Vector) and 0.5 µm laser sections were obtained and examined through the Carl Zeiss LMS 700 confocal microscope and processed with ZEN 2009 Light Edition Software (Zeiss). To evaluate the co-localization between molecules, Pearson’s coefficients were obtained from at least 30 confocal images using the ImageJ 1.45v software and the JACoP plugin.

### 2.7 Secretion assays

Trophozoites (3x10^6^) in basal conditions or after being incubated with RBCs for 2 min as previously described (Galindo et al., 2021), were washed three times in PBS and incubated with 200 μl of PBS supplemented with 1 mg/ml of E64 (Sigma) and a protease inhibitor cocktail (Roche) for 2 h at 37°C. The samples were centrifuged at 13,000 x g for 10 min to obtain the secretion products (SP) in the supernatant fraction. The trophozoites in the pellet were lysed in the presence of protease inhibitors as previously reported (Bolaños et al., 2016; Galindo et al., 2022) to obtain the trophozoite extracts (TE). Samples were submitted to Western blot assays using α-EhVps35, α-EhVps23, α-EhCP112 or α-actin antibodies as described above. For further experiments, secretion products were processed to purify the extracellular vesicles (EVs), as described below.

### 2.8 Western blot experiments

Trophozoites lysates were obtained in the presence of protease inhibitors (PHMB 10 mM, E-64 10 μg/ml, and a protease inhibitor cocktail). Samples were electrophoresed in 10% SDS-PAGE, transferred to nitrocellulose membranes, and probed with α-EhVps35 (1:500), α-actin (1:3000), α- CP112 (1:3000), α-EhVps23 (1:500), α-EhTom1 (1:500), α-EhVps36 (1:500), α-EhADH (1:500), or α-EhVps32 (1:500) antibodies. Membranes were washed, incubated with the respective HRP-labeled secondary antibodies (Sigma, 1:1 000) according to the species, and revealed with the ECL Prime detection reagent (GE-Healthcare, Chicago, IL, USA), according to the manufacturer´s instructions.

### 2.9 Purification of extracellular vesicles by differential centrifugation

The secretion products of trophozoites were processed to purify the extracellular vesicles (Théry et al., 2006; Galindo et al., 2022). Briefly, the secretion products were centrifuged at 10,000 x g for 30 min to remove cell debris. The final supernatant was then ultracentrifuged at 100,000 × g for 70 min to pellet the small vesicles. The pellet was washed in one volume of PBS to remove contaminating proteins and centrifuged at the same high speed. The purified samples were analyzed by transmission electron microscopy (TEM).

### 2.10 Transmission electron microscopy

EVs purified from trophozoites in basal conditions were prepared for TEM as previously described (Mancilla-Olea et al., 2018). Briefly, the preparations were fixed with 4% paraformaldehyde and 0.5% glutaraldehyde in PBS for 1 h at room temperature, washed with PBS, and dehydrated with increasing concentrations of ethanol. After infiltration, samples were embedded in LR White resin (London Resin Co) and polymerized at 56°C ON to obtain thin sections (60 nm) that were mounted on Formvar‐covered nickel grids followed by ON incubation at 4°C with the α‐EhVps35, α‐EhVps23, or α‐EhADH antibodies (1:50). The samples were then incubated ON with the corresponding gold-labeled secondary antibodies (1:50) according to the specific species: α-mouse IgG was conjugated with 20 nm or 30 nm gold particles, α-rat IgG was conjugated with 10 nm gold particles, and α-rabbit IgG was conjugated with 30 nm gold particles (TED Pella Inc). The samples were then contrasted with uranyl acetate and lead citrate and observed through a Joel JEM‐1011 transmission electron microscope.

### 2.11 Protein-protein docking analysis

The predicted and refined 3D structures of the EhVps32 and EhVps23 (Montaño et al., 2017; Galindo et al., 2021) proteins were used for docking analysis as was the 3D structure of the EhVps35 protein (Díaz-Valdez et al., 2024). EhTom1, a 3D model, was obtained from the I-TASSER server using the VHS domain of the Tom1 protein (PDB: ELK1) from Homo sapiens as the template. The 3D structure was refined through 200 ns of MDS by NAMD2.8 (Phillips et al., 2020) with the force field CHARMM36 to create the topologies of the protein (Huang and Mackerell, 2013). The TIP3 model was applied for the water molecules. The system was solvated using the psfgen software in the VMD program (Humphrey et al., 1996). Next, 17,229 water molecules and 6 chlorine ions were added to neutralize the system which was minimized for 10,000 steps, followed by equilibration under constant temperature and pressure (NPT) conditions for 1 ns with the protein and lipid atoms restrained. Afterward, MDS was run for 200 ns, considering EhTom1 a soluble protein, without position restraints under periodic boundary conditions (PBC), and using an NPT ensemble at 310 K and 200 ns of MD simulation. The structures were visualized using the UCSF Chimera software.

The snapshots were obtained using the clustering analysis of 200 ns MDS with the Carma software (Koukos and Glykos, 2013). The protein-protein docking was done employing different conformers with the Cluspro server (Comeau et al., 2004; Kozakov et al., 2013). The conformers with the highest cluster members and the lowest energy were analyzed on the PDBSum server (Laskowski et al., 1997) and the 3D structures visualization was performed by VMD (Humphrey et al., 1996).

### 2.12 dsRNA-based *Ehvps35* gene silencing

The *Ehvps35*-KD gene silencing was performed using the bacteria-expressed double-stranded RNA (dsRNA) that was soaked with the trophozoites as previously described (Solis et al., 2009). Briefly, HT115 bacteria were transformed with *pL4440/Ehvps35* (Díaz-Valdez et al., 2024) and grown at 37°C in LB broth in the presence of ampicillin (100 mg/ml) and tetracycline (10 mg/ml) (Takiff et al., 1989). *Ehvps35*-dsRNA expression was induced by 1 mM isopropyl β-D-1-thiogalactopyranoside (IPTG) ON at 37°C. dsRNA was then isolated from the bacteria using Trizol Reagent (Invitrogen), according to the manufacturer’s recommendations. DNase I (Invitrogen) and RNase A (Ambion) were added to remove ssRNA and dsDNA molecules; *Ehvps35*-dsRNA was washed with isopropanol and ethanol, analyzed by agarose gel electrophoresis, and its concentration was determined by spectrophotometry. Finally, trophozoites (3 x 10^4^) in TYI-S-33 medium were incubated with purified *Ehvps35*-dsRNA molecules to a final concentration of 5 μg/ml, and the cultures were left for 48 h at 37°C, the time at which we previously determined the silencing effect. Cells growing under standard conditions (without dsRNA) were used as controls.

### 2.13 Migration assays

Serum-starved (3 h) trophozoites (7.5 x 10^4^) were placed in the upper chamber of Transwell inserts (5 μm pore size, 24 well, Costar) and 500 μl of bovine serum was added to the lower chamber. Trophozoites were incubated for 3 h at 37°C and trophozoite migration was determined by counting the number of cells in the lower chamber of the Transwell (Bolaños et al., 2016).

### 2.14 *In vivo* virulence experiments

Four-week-old male hamsters (*Mesocricetus auratus*) weighing 40 ± 5g were fasted for 24 h prior to surgery (Galindo et al., 2022). Subsequently, they were anesthetized with 3% isoflurane and anesthetized again with 1.5% of the same anesthetic during the surgical procedure. The abdominal surfaces of the hamsters were shave, and a longitudinal incision of the abdominal wall was made, to expose the port vein and livers. Subsequently, 1.5×10^6^ trophozoites in 200 μl TYI-S-33 without bovine serum were intraportally inoculated into the animals. The hamsters were sacrificed with an overdose of anesthetic 7 days after the challenge; the whole liver was weighed, and the liver lesion was dissected and weighed to calculate the percentage of damaged tissue in relation to the total liver weight (Pais-Morales et al., 2016; Galindo et al., 2022).

### 2.15 Statistical analysis

Values for all experiments were expressed as the mean and standard error of at least three independent assays carried out by duplicate. Statistical analyses were done with the GraphPad Prism v5.01 software by a paired Student’s t test. *p< 0.05; **p< 0.01, and ***p< 0.001.

### 2.16 Ethics statement

CINVESTAV fulfills the standard of the Mexican Official Norm (NOM-062-ZOO-1999) “Technical Specifications for the Care and Use of Laboratory Animals”, based on the Guide for the Care and Use of Laboratory Animals (“The Guide,” 2011, NRC, USA with the Federal Register Number BOO.02.03.02.01.908), awarded by the National Service for Agrifood Health, Safety and Quality (SENASICA). This organization verifies the state of compliance of such NOM in Mexico and belongs to the Ministry of Agriculture and Rural Development. The Institutional Committee for Animal Care and Use (IACUC/Ethics committee) from CINVESTAV, the regulatory office for research protocols approval involving the use of laboratory animals, reviewed and approved all animal experiments (Protocol Number 0505-12, CICUAL 001).

## 3 Results

### 3.1 Interaction of EhVps35 with secretion-, motility-, and phagocytosis-related proteins

**Figure 1:**
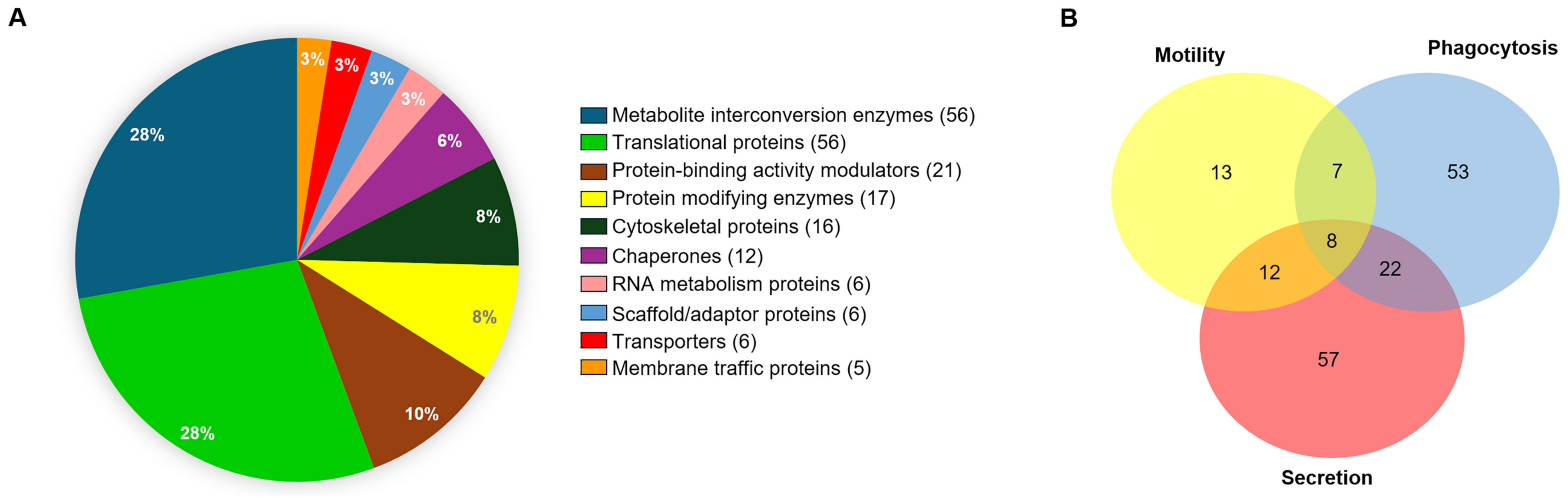
Analysis by mass spectrometry of α-EhVps35 immunoprecipitated trophozoite proteins. **(A)** Proteins detected in the EhVps35 interactome according to the predicted molecular functions using the PANTHER server. Numbers in graph: percentage of proteins. On the right: List of proteins according to their function; In parentheses, the number of proteins found for each function. **(B)** Proteins involved in phagocytosis, motility, and secretion, as described in **Supplementary Tables S2**–**S4**. Numbers indicate the proteins found for each event.

We continued with the characterization of the C isoform of the EhVps35 protein (hereinafter EhVps35) in virulence mechanisms. We investigated by immunoprecipitation experiments and mass spectrometry analyses the proteins that interact with EhVps35, using α-EhVps35 antibodies (Díaz-Valdez et al., 2024). After immunoprecipitation, the proteins were separated by SDS-PAGE, examined by mass spectrometry, and classified according to their function, as described in the literature. The results of the analysis revealed the presence of 300 proteins, from which we selected those related to vesicular trafficking (13 proteins) (**Supplementary Table S1**), motility (40 proteins) (**Supplementary Table S2**), phagocytosis (90 proteins) (**Supplementary Table S3**), and secretion (99 proteins) (**Supplementary Table S4**) (**Figures 1A, B** and **Supplementary Figure S1**). Some of these proteins participate in more than one function (**Figure 1B**), the majority of them (33%) were related to the secretion process in *E. histolytica* trophozoites.

### 3.2 EhVps35 is secreted in extracellular vesicles

**Figure 2:**
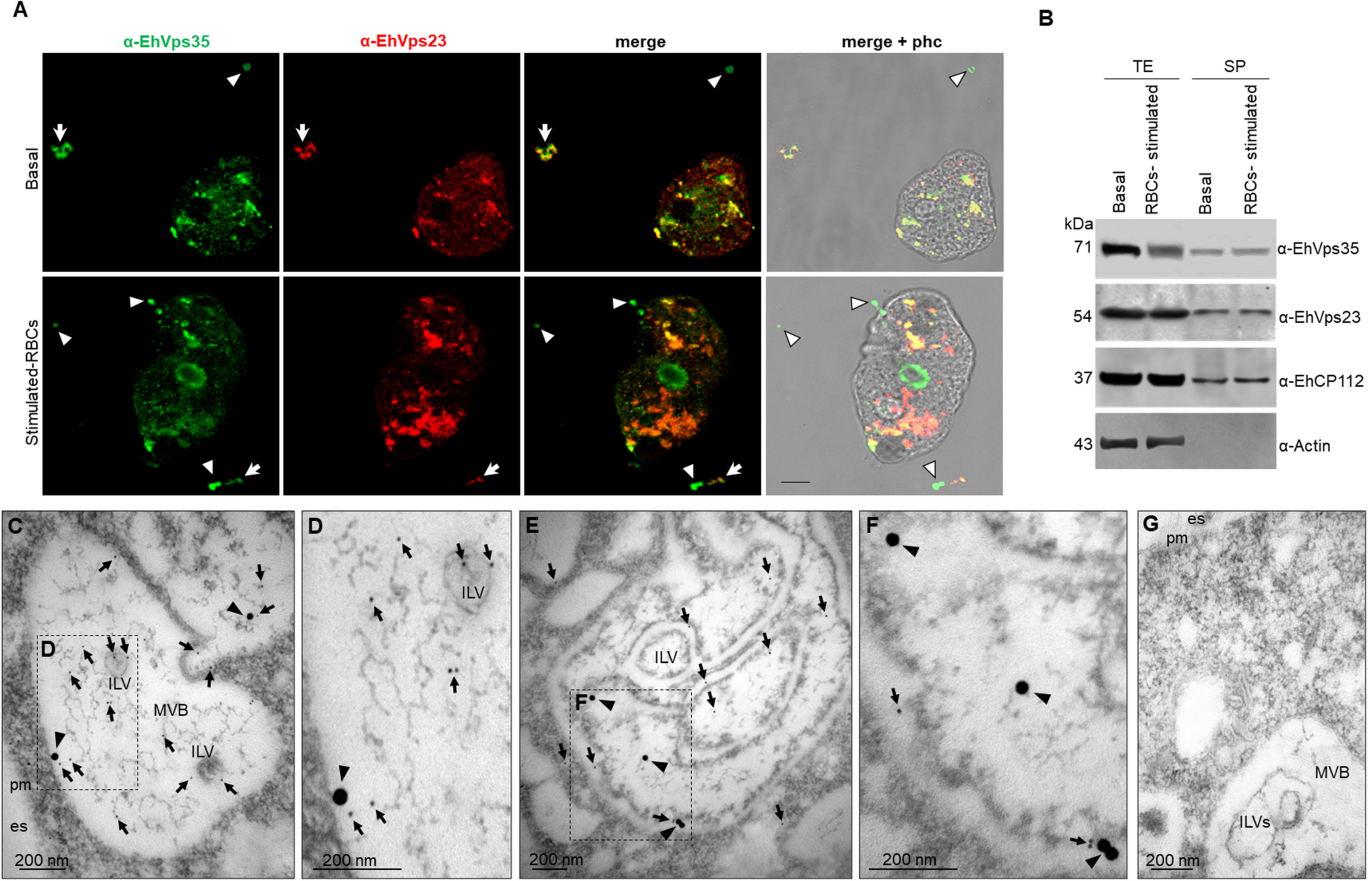
EhVps35 and EhVps23 secretion in basal conditions and under RBCs-stimulus. **(A)** Confocal microscopy images of trophozoites in basal conditions (upper panel) and after 30 min of phagocytosis (lower panel), using α-EhVps35 (green) and α-EhVps23 (red) antibodies. Arrowheads, extracellular structures labeled only with EhVps35. Arrows, extracellular structures labeled with EhVps35 and EhVps23. Scale bar = 10 μm. **(B)** Western blot of trophozoite extracts (TE) and their secretion products (SP) obtained as described in the material and methods section, from trophozoites in basal conditions (basal) and stimulated with RBCs (RBCs-stimulus), revealed with α-EhVps35, α-EhVps23, α-EhCP112, and α-actin. Positive control of secretion: α-EhCP112. Negative control of trophozoite integrity: α-actin. **(C–F)** TEM of thin sections of trophozoites treated with α-EhVps35, and α-EhVps23, and then with gold-labeled α-mouse IgG and α-rat IgG secondary antibodies (30 and 10 nm gold particles, respectively). Squares in C and E are magnified to the right of each image **(D, F)**. es, extracellular space. pm, plasma membrane. ILVs, intraluminal vesicles. MVBs, multivesicular bodies. Arrowheads, EhVps35. Arrows, EhVps23. **(G)** Negative control using only secondary antibodies. Scale: 200 nm.

Next, we explored the mechanism used by the trophozoites to secrete EhVps35, the effect of RBCs-stimulus in the protein secretion, and its relationship with ESCRT machinery proteins. Confocal images of trophozoites in basal and phagocytic conditions evidenced that EhVps35 appeared together with EhVps23 in cytoplasmic and extracellular vesicles. EhVps23 has been reported as an EVs marker in *E. histolytica* (Galindo et al., 2022) (**Figure 2A**). We then analyzed by SDS-PAGE the supernatants with the secreted proteins that were transferred to nitrocellulose membranes and probed with α-EhVps35, α-EhVps23, α-EhCP112, and α-actin, and with the corresponding secondary antibodies (**Figure 2B**). As we previously reported, the amount of EhVps35 protein decreased between 20% and 40% after the RBCs-stimulus to the trophozoites (Díaz-Valdez et al., 2024). Our results confirmed that this reduction remained after 2 h, the time required for the secretion assays. The decrease of EhVp35 in total extracts is possibly due to an increase in secretion (**Figure 2B**). However, we cannot rule out partial degradation of EhVps35 during the assays. In these experiments, the α-EhVps23 antibody served as an internal control (Galindo et al., 2022), the α-EhCP112 as a positive control for secretion (Bolaños et al., 2016), while the α-actin antibody, used as an internal negative control, indicates the integrity of the trophozoites during the experiments (**Figure 2B**) (Bolaños et al., 2016; Galindo et al., 2022). TEM confirmed EhVps35 and EhVp23 localization in MVBs, formed through the ESCRT machinery (**Figures 2C–G**). These structures contain intraluminal vesicles, which are released to generate exosomes.

**Figure 3:**
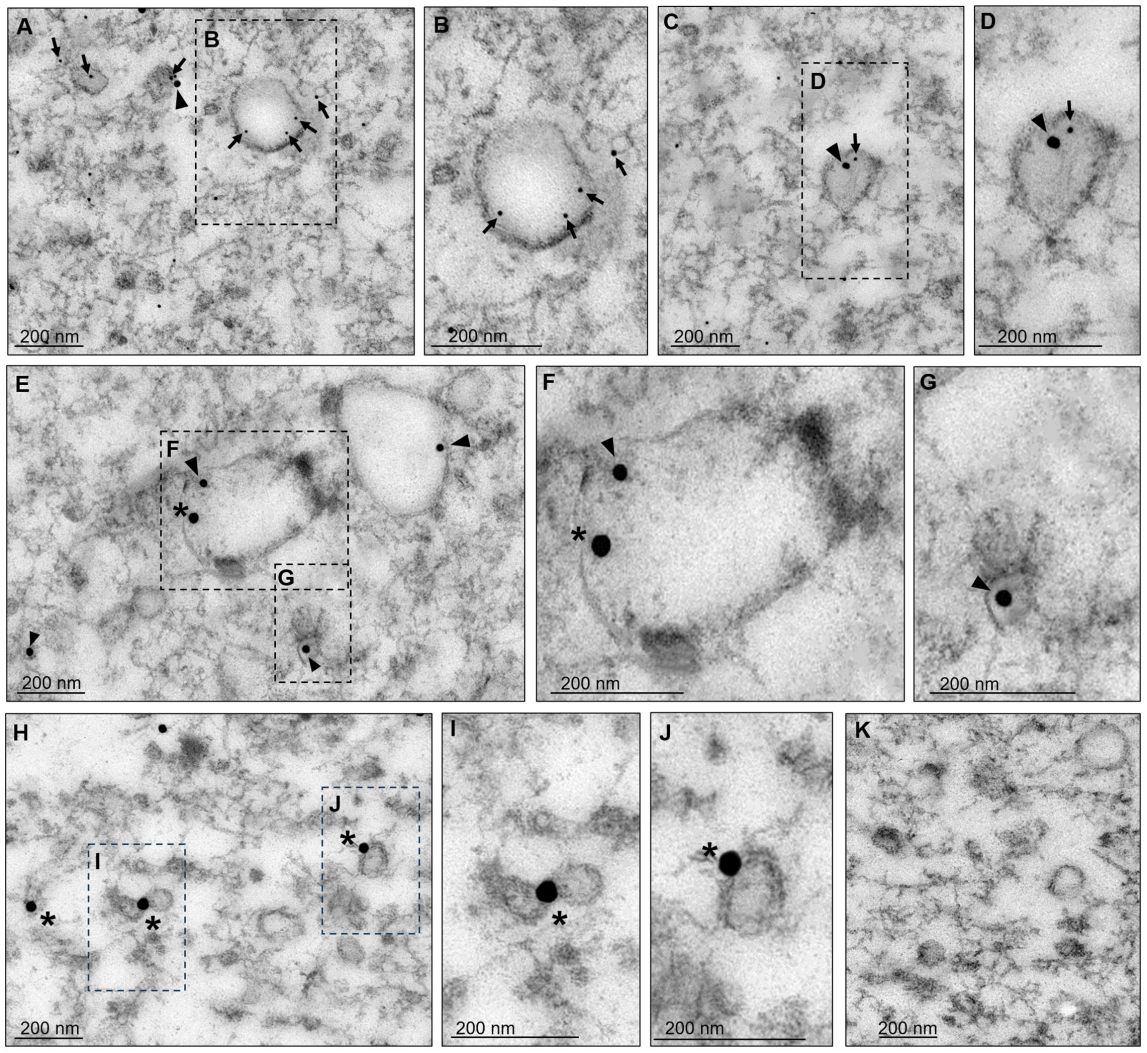
Transmission electron microscopy of EVs found in secretion products. After 2 hours of incubation at 37°C, the supernatants of trophozoites were collected and ultracentrifuged as described in the material and methods section. The pellets were processed for TEM. **(A–J)** Preparations were labeled with α-EhVps35, α-EhVps23, or α-EhADH, and then with gold-labeled α-mouse IgG, α-rat IgG, and α-rabbit IgG secondary antibodies (20, 10, and 30 nm gold particles, respectively). Squares in **(A, C, E, H)** are magnified to the right of each image **(B, D, F, G, I, J)**. Arrowheads, EhVps35. Arrows: EhVps23. Asterisks, EhADH. **(K)** Negative control using only secondary antibodies. Scale: 200 nm.

We proceeded to purify the EVs from the supernatant as described in the materials and methods section to analyze them by TEM. As previously reported (Galindo et al., 2022), the EVs presented in sizes from 32 to 297 nm (**Figure 3**). Thin sections of the EVs were then labeled with α-EhVps23, α-EhVps35, and α-EhADH antibodies and the corresponding secondary antibodies conjugated to gold particles, namely, EhVps35 (20 nm), EhVps23 (10 nm), and EhADH (30 nm), and the samples were then examined by TEM. The EhVps35 protein was located near vesicle membranes and inside EVs (**Figures 3A, C, E**). Recently, Galindo et al. (2022) reported that EhVps23 was also found in EVs, determining that this protein is a key factor in *E. histolytica* secretion, as has also been reported for human cells (Anand et al., 2019). In addition, PDCD61P, an ALIX family member, has been considered an exosome marker in humans (Anand et al., 2019). Thus, we also looked for EhADH in EVs. The images showed that the three proteins appeared in the vesicular membranes and inside them (**Figures 3A–J**). In some vesicles, EhVps35 and EhVps23 (**Figures 3A–D**) or EhVps35 and EhADH (**Figures 3E–G**) were observed together. EhADH was also localized alone in certain vesicles (**Figures 3H–J**), however, it being found alone in vesicles or their solitude may be due to the thin section observed. Interestingly, this is the first time that EhADH has been found in EVs, as has been reported for other ALIX family members. Therefore, our results highlight the importance of secretion for cellular communication and virulence processes (van Niel et al., 2022), suggesting that the EhVps35, EhVps23, and EhADH proteins in EVs might have a role in the *E. histolytica* attack on the host cell. Due to EhVps23 and EhVps35 co-localization in EVs and in the cytoplasm of trophozoites (**Figure 2A**), we proceeded to analyze the interaction between EhVps35 with different proteins of the ESCRT machinery.

### 3.3 EhVps35 and ESCRT protein interactions

**Figure 4:**
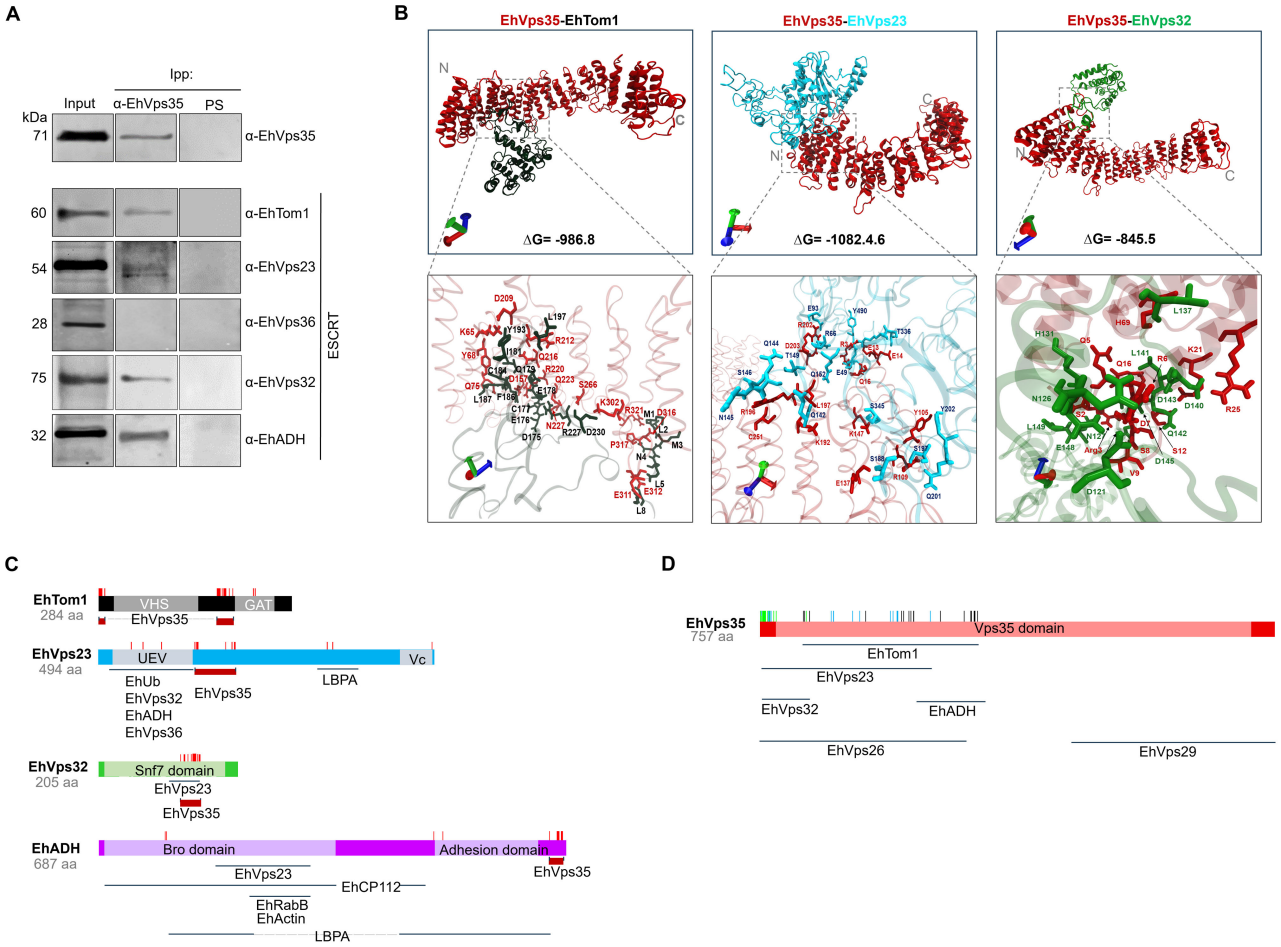
Immunoprecipitation assays and molecular docking of EhVps35/EhTom1, EhVps35/EhVps23, and EhVps35/EhVps32. **(A)** HM1 trophozoites in basal conditions were lysed and immunoprecipitated using the α-EhVps35 antibody. Immunoprecipitated proteins were analyzed by Western blot using the α-EhVps35, α-EhTom1, α-EhVps23, α-EhVps36, α-EhVps32, and α-EhADH antibodies. PS: pre-immune serum used as negative control. **(B)** Molecular docking of EhVps35 (red)/EhTom1 (black), EhVps35 (red)/EhVps23 (blue), and EhVps35 (red)/EhVps32 (green). The lower panel shows the magnification of the interaction sites. **(C)** Schematic representation of the EhTom1, EhVps23, EhVps32 and EhADH proteins with their respective domains, and the molecules that interact with other molecules, as reported in the literature. The amino acid residues (red lines) that participate in each protein in their interaction with EhVps35. **(D)** Schematic representation of the EhVps35 (red) protein, showing the EhVps35 domain (lighter red) and the interaction (vertical lines) with EhTom1 (black), EhVps23 (blue), and EhVps32 (green). Additionally, the interaction sites of EhVps35 for EhVps26, EhVps29, and EhADH are marked below the scheme.

To further investigate the interaction between the retromer and the ESCRT machinery components, we performed immunoprecipitation assays using α-EhVps35, and we searched for components of the different ESCRT machinery subcomplexes using α-EhTom1 (ESCRT-0), α-EhVps23 (ESCRT-I), α-EhVps36 (ESCRT-II), α-EhVps32 (ESCRT-III), and α-EhADH (an ESCRT accessory protein). Western blot assays of immunoprecipitates evidenced that α-EhTom1, α-EhVps23, α-EhVps32, and α-EhADH specific antibodies recognized 60, 54, 32, and 75 kDa bands, respectively, in concordance with previous reports on the migration of each protein (Arroyo and Orozco, 1987; Avalos-Padilla et al., 2015; Galindo et al., 2021; Galindo-Olea, 2022). However, the 28 kDa band that corresponds to EhVps36 did not appear in the immunoprecipitates (**Figure 4A**). This is probably because EhVps36 is an atypical protein, lacking the ubiquitin-binding domain necessary in many systems for the recruitment of other ESCRT machinery proteins (Díaz-Hernández et al., 2023). However, we cannot rule out a very fast dynamic interaction between EhVps35 and EhVps36, since the ESCRT machinery proteins participate in highly dynamic cellular processes with very fast direct or indirect interactions (Avalos-Padilla et al., 2018). Furthermore, as an interaction between EhVps36 and EhVps23 has been previously reported (Díaz-Hernández et al., 2023), an indirect interaction between EhVps35-EhVps36 could be mediated by EhVps23, a highly labile protein that is frequently degraded during experimental procedures (Galindo et al., 2022), making it difficult to study its interactions.

To strengthen the evidence of the association among the ESCRT machinery components and the retromer, we performed docking analysis using 3D models of the proteins already reported (Galindo et al., 2021; Díaz-Valdez et al., 2024) and the EhTom1 3D model was obtained using the I-TASSER server. The global free energy of the EhVps35-EhTom1 interaction was calculated as ΔG = -986.8 (**Figure 4B**). The EhVps35 predicted residues that interacted with EhTom1 were K65, Y68, Q75, D157, D209, R212, Q216, R220, Q223, N227, S266, K302, E311, E312, D316, P317, and R321. Meanwhile, in EhTom1, there were M1, L2, M3, N4, L5, L8, D175, E176, C177, E178, Q179, I181, C184, Q185, F186, L187, Y193, L197, R227, and D230. R227 and D230 were located in the EhTom1GAT domain (**Figures 4B, C**), a ubiquitin-binding domain (Bañuelos et al., 2022). The majority of the EhTom1 residues that interacted with EhVps35 were located between the VHS and GAT domains (**Figure 4C**), suggesting that the binding of EhVps35-EhTom1 does not interfere with EhTom1 binding to other proteins.

The docking analysis also predicted that EhVps35 interacts with EhVps23 with an overall free energy of ΔG = -1082.46 (**Figure 4B**). The EhVps35 predicted residues that interact with EhVps23 were R3, E13, E14, Q16, Y105, R109, E137, K147, K192, L197, R196, R202, D203, and C251; while in EhVps23 they were E49, R66, E93, Q142, Q144, N145, S146, T149, Q152, S188, S197, Q201, Y202, T336, S345, and Y490. Only three residues appeared in the EhVps23 UEV domain (E49, R66, and E94), and one residue (Y490) in the Vps23 core domain. The majority of the EhVps23 predicted residues were located between the UEV and Vps23 core domains, suggesting that EhVps23-EhVps35 binding does not interfere with EhVps23 binding to other ESCRT proteins and other molecules such as EhUbiquitin (EhUb) and LBPA (Galindo et al., 2021) (**Figure 4C**). The EhVps23 UEV domain participates in the binding to EhUb, EhVps36, EhVps32, and EhADH (Galindo et al., 2021; Díaz-Hernández et al., 2023); meanwhile, in other organisms, the Vps23 core domain is responsible for congregating other ESCRT proteins (Kostelansky et al., 2007).

The EhVps35-EhVps32 interaction showed a global free energy of ΔG = -845.5 (**Figure 4B**). The predicted residues of EhVps35 interacting with EhVps32 were S2, R3, Q5, R6, D7, S8, V9, S12, Q16, K21, R25, and H69; while in EhVps32, they were D121, N126, N127, H131, L137, E139, D140, L141, Q142, D143, D145, E148, and L149, located in the EhVps32 Snf7 domain (**Figure 4C**). The majority of the EhVps32 residues (D121, N127, H131, L137, E139, D140, L141, Q142, D145, and E148) are in the same site as those that interact with EhVps23 (Galindo et al., 2021), suggesting that the EhVps35-EhVps32 interaction prevents the EhVps23-EhVps32 union, or that a fast connection of these pair proteins occurs at a certain time. The amino acid location of the selected ESCRT proteins to predict their binding to EhVps35 are depicted in the scheme in **Figures 4C, D**. Some data were obtained from our docking analysis and other data from reports in the literature (Nakada-Tsukui et al., 2005; Castellanos-Castro et al., 2016; Montaño et al., 2017; Srivastava et al., 2017; Galindo et al., 2021; Díaz-Hernández et al., 2023; Díaz-Valdez et al., 2024).

These results strongly suggest an association of the retromer proteins with the ESCRT machinery as shown in the scheme depicted in **Figures 4C, D**. It has already been reported that several of these proteins are involved in phagocytosis (Arroyo and Orozco, 1987; García-Rivera et al., 1999; Avalos-Padilla et al., 2015, 2018; Galindo et al., 2021) and in other virulence events of *E. histolytica* (Galindo et al., 2022).

### 3.4 *Ehvps35* gene knockdown (*Ehvps35*-KD) alters the cellular location of some ESCRT machinery components

**Figure 5:**
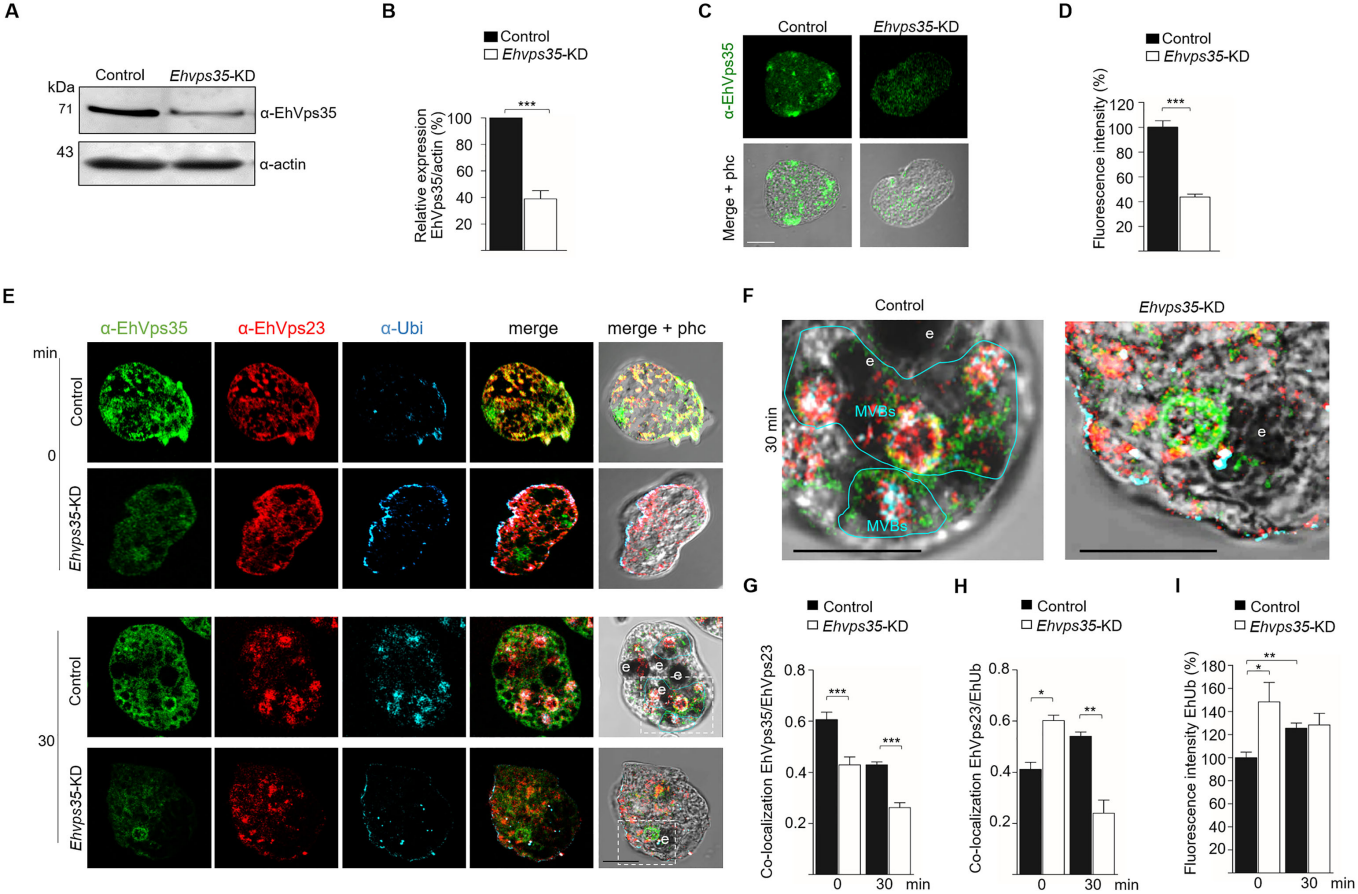
*Ehvps35*-KD trophozoites present alterations in EhVps23 and EhUb cellular locations. **(A)** Western blot assays of non-silenced (control) and silenced (*Ehvps35*-KD) trophozoites. **(B)** Densitometric analysis of the bands shown in **(A)** normalized against actin. **(C)** Confocal microscopy representative images of control and *Ehvps35*-KD trophozoites under basal conditions using the α-EhVps35 antibody. **(D)** Fluorescence intensity of the images in **(C)** measured in pixels. **(E)** Trophozoites in basal conditions (0 min) and stimulated with RBCs (30 min) were processed for immunofluorescence using α-EhVps35 (green), α-EhVps23 (red), and α-Ub (blue) antibodies. **(F)** Zoom: Magnification of regions marked by squares in merged images **(E)**. Scale bar, 10 μm. e, erythrocytes. MVBs, multivesicular bodies (marked by a blue line). **(G, H)** Pearson’s coefficient for EhVps35/EhVps23 and EhVps35/EhUb co-localization in control and Ehvps35-KD trophozoites. I) Fluorescence intensity of EhUb measured in pixels. *p < 0.05, **p < 0.01, ***p < 0.001.

We have recently reported that the *Ehvps35*-KD gene alters the trophozoite localization of EhADH (Díaz-Valdez et al., 2024). To study the effect of *Ehvps35*-KD trophozoites on EhVps23 and EhVps32, we obtained *Ehvps35*-KD trophozoites as previously described (Díaz-Valdez et al., 2024). In concordance with previous experiments (Díaz-Valdez et al., 2024), the Western blot and confocal immunofluorescence assays evidenced a 60% reduction in the EhVps35 protein expression in the newly obtained *Ehvps35*-KD trophozoites (**Figures 5A–D**). To explore the impact of *Ehvps35*-KD on the localization and function of the EhVps23 and EhVps32 proteins, we performed pulse-chase erythrophagocytosis assays as described in the materials and methods section. After 30 min of phagocytosis, we searched, by confocal microscopy, for the EhVps35 and EhVps23 proteins, using the α-EhVps35 and α-EhVps23 antibodies. We also analyzed the EhUb localization because EhVps23 frequently binds to ubiquitinated proteins for the recruitment of other ESCRT machinery components (Galindo et al., 2021). The results obtained using non-silenced (control) trophozoites in basal conditions showed a poor signal for α-Ub, whereas α-*EhVps35* and α-EhVps23 recognized small spots in the plasma membrane and cytoplasmic vesicular structures. The antibodies co-localized the EhVps35, EhVps23, and EhUb proteins at discrete points in the plasma membrane, but poor co-localization was detected in the cytoplasm. However, EhVps35 and EhVps23 were co-localized in dots and in circular structures (**Figure 5E**) while EhVps23 and EhUb appeared as small points near the plasma membrane, as described (Galindo et al., 2021). In contrast, in *Ehvps35*-KD trophozoites, EhVps35 exhibited a low signal, while the localization of EhVps23 increased in the plasma membrane where it mostly co-localized with EhUb (**Figure 5E**). After 30 min of phagocytosis, the cellular location of these proteins changed. EhVps35, EhVps23, and EhUb appeared in the cytoplasm in non-silenced trophozoites, co-localizing around the phagosomes. In addition, the α-Ub signal was augmented, possibly due to the ubiquitination of several proteins involved in different steps of phagocytosis. We also observed the co-localization of the three proteins in structures that could correspond to MVBs (**Figures 5E, F**). In contrast, in *Ehvps35*-KD trophozoites, the EhVps35, EhVps23, and EhUb proteins were poorly co-localized and only appeared in small vesicular structures (**Figures 5E–H**). It is worth mentioning that EhUb recognition by EhVps23 initiates the ESCRT machinery component recruitment for MVBs formation (Galindo et al., 2021). Our data evidenced that *Ehvps35* gene silencing affects the EhUb location and fluorescence intensity (**Figures 5E, I**) and it alters both the localization and recruitment of other members of the ESCRT machinery components and the subsequent MVBs formation.

**Figure 6:**
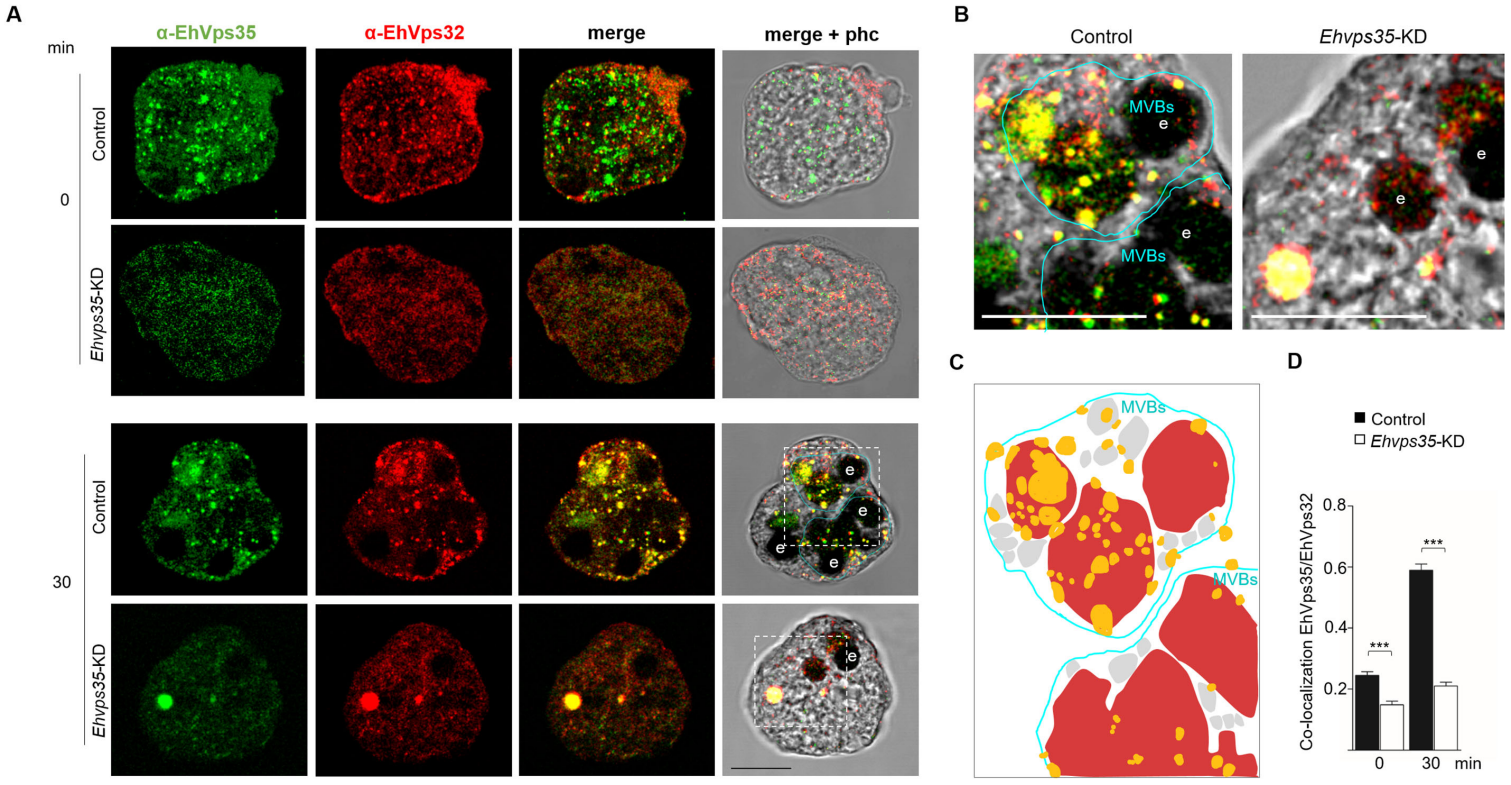
*Ehvps35*-KD trophozoites present alterations in MVBs formation. **(A)** Trophozoites in basal conditions (0 min) and stimulated with RBCs (30 min) were processed for immunofluorescence using α-EhVps35 (green) and α-EhVps32 (red) antibodies in non-silenced: (control) and silenced (*Ehvps35*-KD) trophozoites. **(B)** Zoom: Magnification of regions marked by squares in merged images. e: erythrocytes. MVBs: multivesicular bodies. Scale bar = 10 μm. **(C)** Schematic representation of the confocal image (**(B)**, left panel) showing. MVBs (blue line) surrounded by multiple vesicular structures (gray) in control trophozoites stimulated with RBCs (red). Yellow: EhVps35 and EhVps32 co-localization. **(D)** Pearson’s coefficient for EhVps35-EhVps32 co-localization. ***p < 0.001.

The effect of *Ehvps35*-KD on the location of EhVps23 and EhUb could also impact EhVps32 cellular localization, an ESCRT-III component that regulates the intraluminal vesicles (ILVs) (Avalos-Padilla et al., 2018). We proceeded to analyze, by confocal microscopy, whether the cellular location of EhVps32 was also affected in *Ehvps35*-KD trophozoites. Our results showed no co-localization of EhVps35 and EhVps32 in basal conditions. Both proteins appeared in vesicular structures in the cytoplasm, and, in some cells, EhVps32 presented polarization to a membrane pole. In *Ehvps35*-KD trophozoites, fluorescence staining was poorly observed with the α-EhVps35 antibody, and the EhVps32 protein appeared dispersed in the cytoplasm (**Figure 6A**). Interestingly, after 30 min of erythrophagocytosis in the control trophozoites, EhVps35 and EhVps32 appeared together in MVBs (**Figures 6A–D**). In contrast, in *Ehvps35*-KD trophozoites, EhVps35 and EhVps32 were co-localized only in cytoplasmic vacuoles, and no MVBs were visualized (**Figures 6A, D**). Previous studies in human cells have reported that *Hsvps35* gene silencing causes an alteration in the ESCRT machinery functions (Filippone et al., 2021a; Walsh et al., 2021; Tan et al., 2022). Our results suggest that in *E. histolytica*, EhVps35 also indirectly participates in the recruitment of the ESCRT machinery, because in *Ehvps35*-KD trophozoites, EhUb (a key initiator molecule for this process) had an aberrant cellular location. In addition, from our results, it was logical to assume that these alterations affect virulence processes in the parasite.

### 3.5 Migration is affected in *Ehvps35*-KD trophozoites

**Figure 7:**
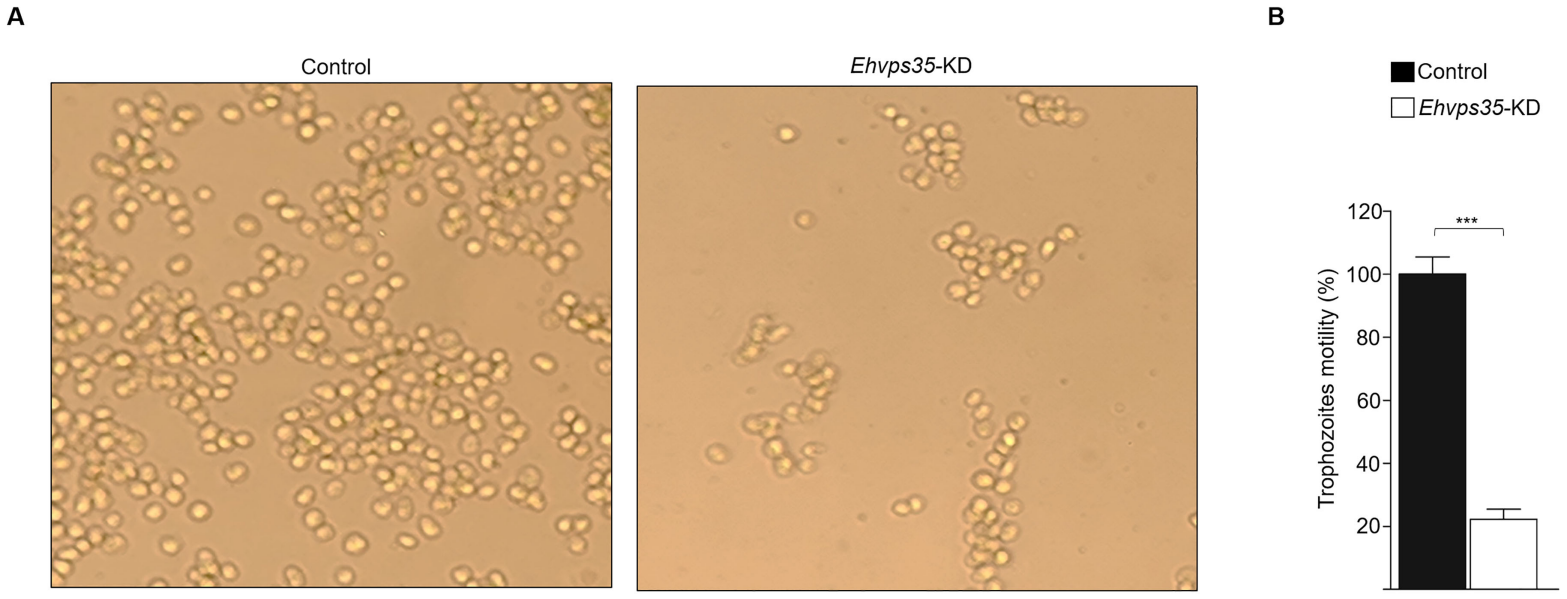
*Ehvps35*-KD trophozoites present a reduction in motility. Non-silenced (control) and *Ehvps35*-KD trophozoites were placed in the upper chamber of Transwell filters with bovine serum in the lower chamber. After 3 h, the trophozoites in the lower chamber were counted. **(A)** Images of control and *Ehvps35*-KD trophozoites in the lower chamber of the Transwell filters. **(B)** Trophozoite quantification in the lower chamber. ***p < 0.001.

EhVps23 is key for trophozoite migration (Galindo et al., 2022) and the experiments performed in this study have shown that the *Ehvps35*-KD gene affects EhVps23 cellular location and MVBs formation. In addition, we have reported that *Ehvps35*-KD causes an alteration in the cytoskeleton, a key element in movement (Díaz-Valdez et al., 2024). Furthermore, there are reports that indicate that, in cancer human cells, Hs*vps35* gene silencing reduces the ability of the cells to migrate (Liu et al., 2012; Tan et al., 2022). We proceeded to investigate whether the *Ehvps35*-KD trophozoites presented with decreased motility, using a chemoattractant in Transwell filters (Bolaños et al., 2016). Trophozoites were placed in the upper chamber of the filters, and the lower chamber was charged with 500 μl of bovine serum. Migration was measured by the ability of the trophozoites to move to the chemoattractant. After 3 h incubation at 37°C, the *Ehvps35*-KD trophozoites showed an 80% decrease in their capacity to migrate toward the chemoattractant compared to the migration of the control trophozoites (**Figures 7A, B**). These results strongly suggest that EhVps35 is strongly involved in the motility signaling pathways involved in migration. This could explain the high number of motility- and cytoskeleton-involved proteins detected in the mass spectrometry analysis presented in **Figure 1**.

### 3.6 *Ehvps35*-KD trophozoites showed decreased *in vivo* virulence

**Figure 8:**
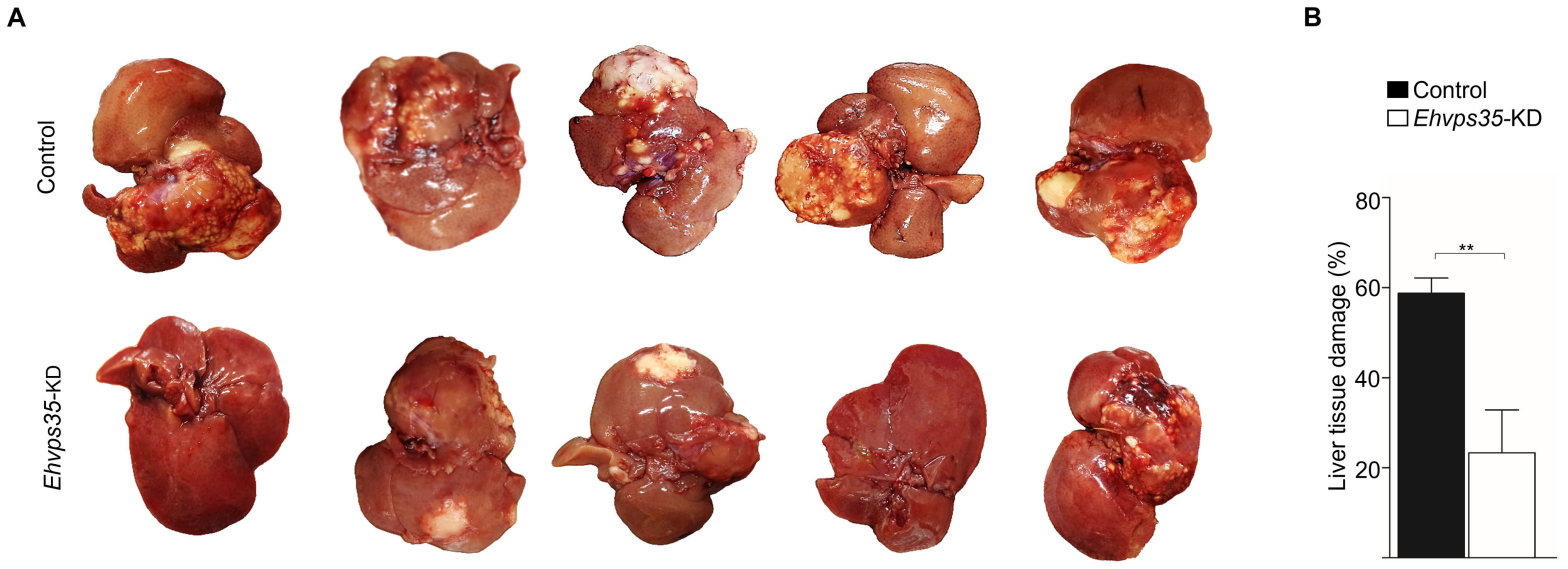
*Ehvps35*-KD trophozoites present a reduction in hepatic abscess formation in hamsters. **(A)** Animals under anesthesia were inoculated with non-silenced (control) and *Ehvps35*-KD trophozoites. The animals were anesthetized 7 days later and their livers were extracted to examine the damage caused by the trophozoites. **(B)** The percentage of the damage was calculated by weighing the livers, weighing the damaged tissue, and obtaining the relation between them. **p < 0.01.

*E. histolytica* movement is a key element for trophozoites to invade and reach different organs such as the liver where they cause hepatic abscesses. We investigated the ability of *Ehvps35*-KD trophozoites to produce hepatic abscesses in hamsters. The animals were anesthetized and intraportally inoculated with either control or *Ehvps35*-KD trophozoites. After 7 days of inoculation, the control animals presented with bristly hair and abdominal inflammation, and appeared depressed, while the animals inoculated with *Ehvps35*-KD trophozoites appeared healthy. Thus, under deep anesthesia, we examined their livers. The results showed that the control trophozoites produced a huge number of abscesses, whereas the *Ehvps35*-KD trophozoites caused only a few small ones (**Figure 8A**). Quantification of the healthy and damaged tissue showed that the control trophozoites damaged approximately 58.8% of the liver tissue, while the hamsters inoculated with the same number of *Ehvps35*-KD trophozoites presented with only approximately 23.3% of the tissue damaged (**Figure 8B**). The decrease in liver damage presented by the *Ehvps35*-KD trophozoites could be related to their motility impairment and to the alteration of the cellular location of EhVps23. It has been reported that when the *Ehvps23* gene is overexpressed, the hepatic abscesses are exacerbated (Galindo et al., 2022). Our results suggest that EhVps35 is a key protein for the correct localization and function of the ESCRT machinery components, and they strengthened the assumption that the relationship between both complexes is crucial for virulence mechanism expression in *E. histolytica*. In addition, they influence other vital functions of trophozoites.

## 4 Discussion

Herein, we studied the relationship of the EhVps35 protein, the central member of the retromer (Nakada-Tsukui et al., 2005; Watanabe et al., 2020), with the ESCRT machinery. We selected EhVps35 for our study because experimental evidence suggests that Vps35 participates in multiple key cellular events involved in pathogenesis in different systems: i) in humans, HsVps35 is a malignant cancer marker since it induces cell growth, motility, and the invasion capacity of cancer cells (Liu et al., 2020; Tan et al., 2022); ii) HsVps35 is also related to EVs formation and secretion (Walsh et al., 2021; Tan et al., 2022); iii) in *E. histolytica*, EhVps35 actively participates in vesicular trafficking, specifically in the recycling of plasma membrane-associated proteins (Díaz-Valdez et al., 2024). Therefore, this protein might be involved in several trophozoite virulence functions (Díaz-Valdez et al., 2024). Knowledge of EhVps35 functions in virulence mechanisms provides an excellent tool for discovering interconnections among proteins and cellular events. Hence, the molecular characterization of this protein could help in the development of strategies for the detection of amoebiasis and treatment designs to combat it.

Our work provides evidence on: i) the involvement of ESCRT machinery and retromer proteins in vesicular trafficking, phagocytosis, secretion, and motility, clue events in virulence mechanisms (**Supplementary Tables S1**–**S4**); ii) the effect of RBCs-stimulus on EhVps35 secretion (**Figure 2**); iii) the presence of EhVps35 together with EhVps23 and EhADH in EVs (**Figure 3**); iv) the interaction of EhVps35 with the Tom1 (ESCRT-0), EhVp23 (ESCRT-I), and EhVps32 (ESCRT-III) proteins (**Figure 4**); v) the low expression of EhVps35 caused changes in the cellular localization of EhVps23 and EhVps32, which negatively impacts MVBs formation (**Figures 5**, **6**); and, interestingly, vi) the reduction in trophozoite motility and hepatic abscess formation in cells poorly expressing EhVps35, suggesting that the retromer is an important player in tissue invasion (**Figures 7**, **8**). In total, 57.3% of the 300 proteins that were detected by mass spectrometry to interact with the EhVps35 protein are related to secretion, motility, and phagocytosis, highlighting the importance of the retromer in these events. All of the events require the main retromer functions: selection, sorting, and recycling.

In humans, TSG101 (Vps23 in other organisms) has been considered an exosome marker (Anand et al., 2019) and Vps35 secretion in EVs has been used a malignant marker in cancer cells and in neurodegenerative diseases, where the EVs secretion is of great importance for intercellular communication and the prognosis of these diseases (Liu et al., 2012; Zhang et al., 2018; Ferraiuolo et al., 2020; Filippone et al., 2021b, 2021a; Walsh et al., 2021; Tan et al., 2022). In fact, there are reports suggesting that HsVps35 impacts EV formation in humans (Gross et al., 2012; Tan et al., 2022). During infection, the *E. histolytica* trophozoites can migrate from the intestine to the liver and other organs in a similar process to the metastasis of cancer cells (Orozco et al., 1994; Leroy et al., 1995). Our results from the immunoprecipitation assays using α-EhVps35 antibody and mass spectrometry, showed that 33% of the identified proteins corresponded to proteins detected by (Sharma et al., 2020) in *E. histolytica* EVs, including EhVps35 (**Figure 1**). EhADH, EhVps23, and EhVps35 were detected in vesicles and could correspond to the exosomes generated as a result of the release of MVBs intraluminal vesicles, formed through the ESCRT machinery. These findings also suggest the participation of the retromer and ESCRT components in intercellular communication in *E. histolytica* trophozoites using EVs as a transportation medium, an event that has been poorly studied in this parasite. Furthermore, a low expression of EhVps35 caused a decrease in the phagocytic capacity of the trophozoites (Díaz-Valdez et al., 2024). At the same time, there was an increase in EhVps35 secretion in the RBCs-stimulus trophozoites, evidencing retromer participation in phagocytosis (**Figures 2**, **3**). Multiple proteins of the ESCRT machinery and the retromer exhibit re-localization to different cellular structures during phagocytosis in trophozoites (Arroyo and Orozco, 1987; García-Rivera et al., 1999; Nakada-Tsukui et al., 2005; Avalos-Padilla et al., 2015, 2018; Ocádiz-Ruiz et al., 2016; Srivastava et al., 2017; Watanabe et al., 2020; Galindo et al., 2021, 2022; Díaz-Hernández et al., 2023; Díaz-Valdez et al., 2024).

We have further analyzed the interaction of EhVps35 with the ESCRT machinery proteins, performing immunoprecipitation assays using the α-EhVps35 antibody, Western blot, and molecular docking analysis. The results showed that EhVps35 interacts with ESCRT-0 (EhTom1), ESCRT-I (EhVps23), ESCRT-III (EhVps32), and ESCRT accessory proteins (EhADH) (**Figure 4**). This assumption is supported by Galindo et al. (2021) results on the interaction between EhVps23-EhVps32 and EhVps23-EhADH. In addition, EhTom1 and EhVps23 have domains that allow interaction with ubiquitinated proteins, an event that initiates the recruitment of other ESCRT machinery components. However, we do not know if these are direct or indirect interactions and we assume that these interactions are highly dynamic and fast as a part of the chain of events presented during phagocytosis.

Furthermore, *Ehvps35*-KD in trophozoites produced an impairment in EhVps23 and EhVps32 cellular localization and a decrease in MVBs formation (**Figures 5**, **6**). These results strongly support that the function of both complexes is codependent, as has been reported for humans and *D. melanogaster* (Dukes et al., 2011; Pannen et al., 2020; Walsh et al., 2021; Tan et al., 2022). Interestingly, low expression of EhVps35 led to the aberrant localization of EhUb and augmentation of fluorescence intensity in basal conditions, which is related to the altered localization of EhVps23 and EhVps32. This agrees with reports in human neuronal cells, where a reduction in HsVps35 expression causes an alteration in the degradation pathways, provoking an accumulation of the ubiquitin mark (Filippone et al., 2021b, 2021a). Our results suggest that in *E. histolytica*, EhVps35 has a role in protein degradation mediated by the ESCRT machinery (Díaz-Valdez et al., 2024).

The HsVps35, TSG101, and CHMP6 (orthologous of EhVps35, EhVps23, and EhVps32) proteins have been widely studied in multiple cellular processes, such as cell migration, secretion, and cell proliferation in cancer (Zhang et al., 2018; Liu et al., 2020; Tan et al., 2022). In addition, several of the *E. histolytica* ESCRT proteins have been related to virulence mechanisms (cell proliferation, secretion, phagocytosis, motility, and hepatic abscess formation) (Arroyo and Orozco, 1987; García-Rivera et al., 1999; Bañuelos et al., 2012; Avalos-Padilla et al., 2015, 2018; Ocádiz-Ruiz et al., 2016; Galindo et al., 2021, 2022; Díaz-Hernández et al., 2023). This is supported by the results obtained using *Ehvps35*-KD trophozoites, where we observed a reduction in their motility and in their ability to cause hepatic abscesses, strengthening the presumption that this protein plays a key role in the virulence of *E. histolytica* (**Figures 7**, **8**).

In conclusion, our work provides an overview and presents experimental evidence of the complex interactions between EhVps35 and the ESCRT machinery proteins during phagocytosis and protein transport to different cellular compartments in *E. histolytica*, impacting their virulence. This primitive protozoan is an excellent model in which to study vesicular transport to further explore retromer functions and other cellular mechanisms. Furthermore, the impact of EhVps35 on hepatic abscess formation and migration of *E. histolytica* will reveal new targets for anti-amoebiasis drug design.

## Data Availability

The datasets presented in this study can be found in online repositories. The names of the repository/repositories and accession number(s) can be found in the article/**Supplementary Material**.
