# Supplementary material for: EhVps35, a retromer component, is a key factor in secretion, motility, and tissue invasion by Entamoeba histolytica
Source: Front Cell Infect Microbiol. 2024 Sep 27;14:1467440. doi: 10.3389/fcimb.2024.1467440 (PMC11466944; doi:10.3389/fcimb.2024.1467440)
Supplement: Supplementary file 1 [file Table1.docx]

**Supplementary material**


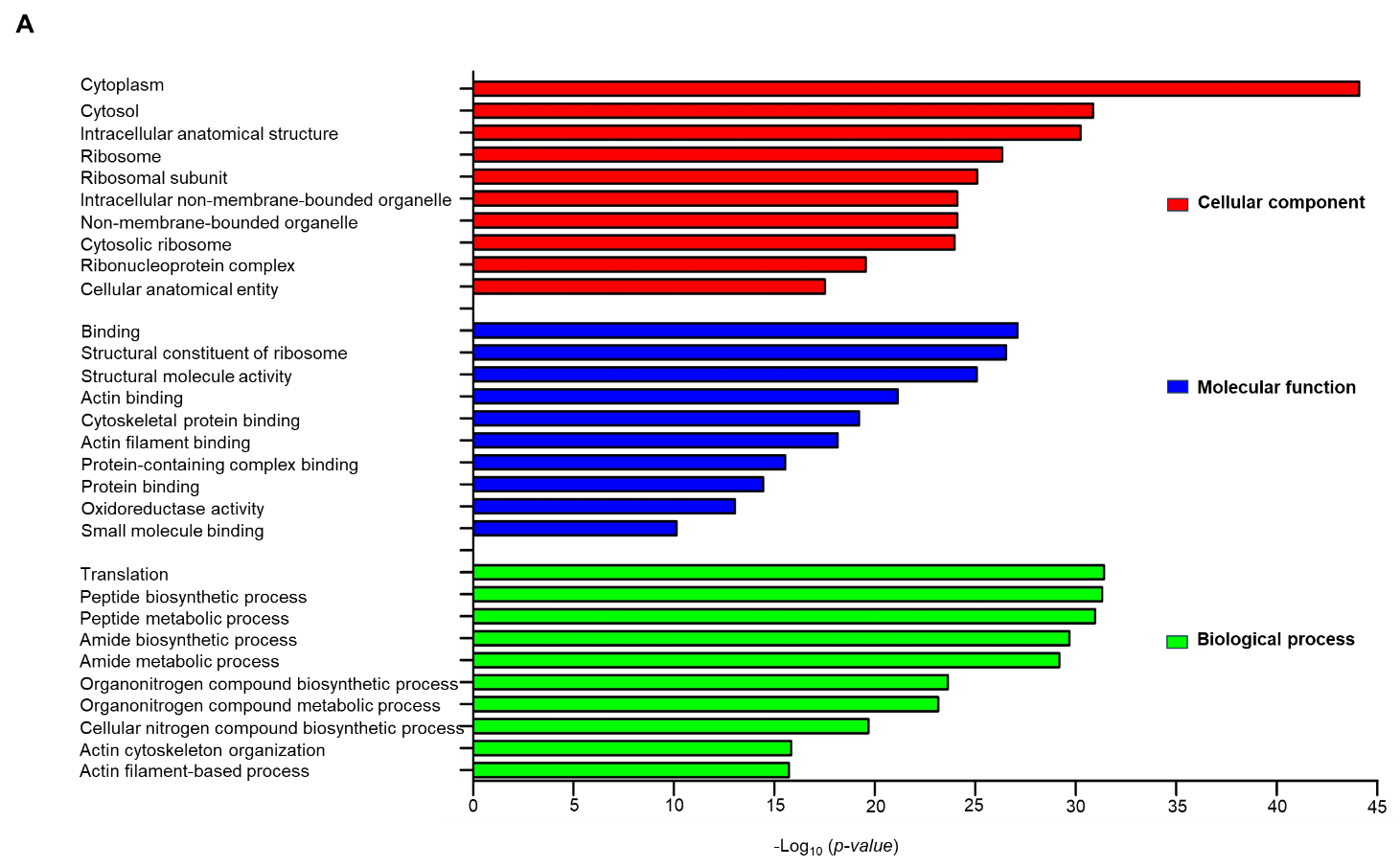


**Figure S1:** Gene ontology (GO) of immunoprecipitated proteins using α-EhVps35 antibody, were carried out using the GeneOntology database (http://geneontology.org/). GO analysis allows the association of a given gene list with specific functional annotations, which are further divided into functional clusters listed according to an enrichment P-value. The 10 most significant enriched GO terms in biological process, molecular function and cellular component branches are presented. All the adjusted statistically significant P-values of the terms were negative 10-base log normalized.

| Table S1. Proteins of vesicular trafficking. | |
| --- | --- |
| Protein | **Access number** |
| AP-1 complex subunit mu 2 | EHI_089880 |
| AP-2 complex subunit mu | EHI_124560 |
| Vacuolar protein sorting-associated protein | EHI_012580 |
| Rab5 | EHI_026420 |
| Rab11D | EHI_056100 |
| Rab2C | EHI_067850 |
| Rab7B | EHI_081330 |
| RabC1 | EHI_153690 |
| RabD2 | EHI_164900 |
| Rab11B | EHI_107250 |
| Vacuolar sorting protein 26 | EHI_008470/EHI_062490 |
| Vacuolar sorting protein 35 | EHI_002990 |

| Table S2. Proteins associated with motility *. | |  |
| --- | --- | --- |
| Protein | **Access number** |  |
| Beta-hexosaminidase subunit beta | EHI_007330 |  |
| Pyruvate phosphate dikinase | EHI_009530 |  |
| Cysteine proteinase 2 | EHI_033710 |  |
| RabX31 | EHI_040310 |  |
| V-type ATPase, A subunit | EHI_043010 |  |
| Actin-related protein 2/3 complex subunit | EHI_045000 |  |
| Heat shock protein 70 | EHI_052860 |  |
| RAP1 | EHI_058090 |  |
| RACC | EHI_070730 |  |
| Protein disulfide isomerase | EHI_071590 |  |
| Steroid 5-alpha reductase | EHI_076870 |  |
| Rab7B | EHI_081330 |  |
| Small ribosomal subunit protein uS2 | EHI_081410 |  |
| Ubiquitin | EHI_083270 |  |
| 4-alpha-glucanotransferase | EHI_084680 |  |
| DEAD/DEAH box helicase | EHI_093900 |  |
| Filamin 2 | EHI_104630 |  |
| Rab11B | EHI_107250 |  |
| Myosin II heavy chain | EHI_110180 |  |
| Grainin | EHI_120360 |  |
| 60S Ribosomal protein L9 | EHI_126140 |  |
| Peptidase-CP-C6 | EHI_127030 |  |
| RACG | EHI_129750 |  |
| Enolase 1 | EHI_130700 |  |
| Protein disulfide-isomerase | EHI_133970 |  |
| Calreticulin | EHI_136160 |  |
| Dipeptidyl-peptidase | EHI_136440 |  |
| RabC3 | EHI_143650 |  |
| Gal/GalNAc lectin light subunit | EHI_148790 |  |
| Alcohol dehydrogenase | EHI_150490 |  |
| RabC1 | EHI_153690 |  |
| 3-ketoacyl-CoA synthase | EHI_158240 |  |
| 90 kDa Heat shock protein | EHI_163480 |  |
| Grainin 1 | EHI_167300 |  |
| Grainin 2 | EHI_167310 |  |
| Cysteine proteinase 5 | EHI_168240 |  |
| RabX11 | EHI_177520 |  |
| V-type ATPase, B subunit | EHI_189850 |  |
| Sulfate adenylyltransferase | EHI_197160 |  |
| 70 kDa Heat shock protein | EHI_199590 |  |
| * Markiewicz et al., 2011. | | |

| Table S3. Proteins associated with phagocytosis **. | |
| --- | --- |
| Protein | **Access number** |
| Calcium-regulated actin-bundling protein C-terminal domain-containing protein | EHI_004550 |
| Hydroxylamine reductase | EHI_004600 |
| TNase-like domain-containing protein | EHI_005150 |
| Beta-hexosaminidase subunit beta | EHI_007330 |
| NECAP PHear domain-containing protein | EHI_007350 |
| Aminopeptidase | EHI_008380 |
| Calcium-regulated actin-bundling protein C-terminal domain-containing protein | EHI_010570 |
| Elongation factor 1α | EHI_011210 |
| 60S ribosomal protein L10a | EHI_012480 |
| Proton-translocating NAD(P)(+) transhydrogenase | EHI_014030 |
| Ribosomal protein L15 | EHI_020300 |
| Uncharacterized protein | EHI_020330 |
| NADP-dependent alcohol dehydrogenase | EHI_023110 |
| Calmodulin | EHI_023500 |
| Cysteine synthase A | EHI_024230 |
| 14-3-3 protein 1 | EHI_025360 |
| PPi-type phosphoenolpyruvate carboxykinase 2 | EHI_030750 |
| GTP-binding protein | EHI_031410 |
| Polyadenylate-binding protein | EHI_033250 |
| Cysteine protease 2 | EHI_033710 |
| Uncharacterized protein | EHI_035730 |
| Rho guanine nucleotide exchange factor | EHI_039480 |
| Aminoacyl-histidine dipeptidase | EHI_042170 |
| 60S ribosomal protein L10 | EHI_044810 |
| Uncharacterized protein | EHI_047800  ^b^ |
| Lysine-tRNA ligase | EHI_047810 |
| Purine nucleoside phosphorylase | EHI_048740 |
| Nitrogen fixation protein NifU | EHI_049620 |
| phosphoglycerate mutase | EHI_050940 |
| Serine carboxypeptidase | EHI_054530 |
| RAP1 | EHI_058090 |
| Vacuolar protein sorting 26 (EhVps26) | EHI_062490 |
| Vacuolar protein sorting 35 (EhVps35) | EHI_002990 |
| Adenosylhomocysteinase | EHI_068250 |
| Inositol-3-phosphate synthase | EHI_070720 |
| RACC | EHI_070730 |
| ADP-ribosylation factor 1 | EHI_073470 |
| Mitogen-activated protein kinase | EHI_073650 |
| Long-chain-fatty-acid--CoA ligase | EHI_079300 |
| START domain-containing protein | EHI_080260 |
| Filopodin | EHI_080740 |
| Rab7B | EHI_081330 |
| Small ribosomal subunit protein uS2 | EHI_081410 |
| Coronin | EHI_083590 |
| Seryl-tRNA synthetase | EHI_092640 |
| DEAD/DEAH box helicase | EHI_093900 |
| 14-3-3 protein 2 | EHI_098280 |
| Phosphotransferase | EHI_098290 |
| Cortexillin | EHI_104560 |
| Rab11B * | EHI_107250 |
| Rab1A * | EHI_108610 |
| LIM zinc finger domain containing protein | EHI_110280 |
| Glutamate synthase beta subunit | EHI_110520 |
| PCTP-like protein | EHI_110720 |
| ADF-H domain-containing protein | EHI_118750 |
| Enhancer binding protein-1 | EHI_121780 |
| AP-2 complex subunit mu | EHI_124560 |
| Peptidyl-prolyl cis-trans isomerase | EHI_125840 |
| RACG | EHI_129750 |
| Purine nucleoside phosphorylase | EHI_130960 |
| Protein disulfide-isomerase | EHI_133970 |
| S-phase kinase-associated protein 1A | EHI_134960 |
| 40S ribosomal protein S17 | EHI_135060 |
| Dipeptidyl-peptidase | EHI_136440 |
| RabC3 | EHI_143650 |
| Rho GDP exchange inhibitor | EHI_147570 |
| RabC1 | EHI_153690 |
| 4Fe-4S ferredoxin-type domain-containing protein | EHI_154450 |
| Thioredoxin reductase | EHI_155440 |
| Proteasome subunit Alpha | EHI_163650 |
| Actinin-like protein | EHI_164430 |
| Alcohol dehydrogenase | EHI_166490 |
| Rab GDP dissociation inhibitor alpha | EHI_167060 |
| Cysteine protease 5 | EHI_168240 |
| Aspartyl-tRNA synthetase | EHI_175050 |
| Profilin | EHI_176140 |
| RabX11 | EHI_177520 |
| Dolichyl-diphosphooligosaccharide-protein glycotransferase | EHI_183010 |
| Eukaryotic translation initiation factor 5A | EHI_186480 |
| Actin-binding protein | EHI_186840 |
| Phosphoglycerate kinase | EHI_188180 |
| Calcium-regulated actin-bundling protein C-terminal domain-containing protein | EHI_189930 |
| Molybdenum cofactor sulfurase putative | EHI_194600 |
| Heat shock protein 90 | EHI_196940 |
| 40S ribosomal protein S10 | EHI_197030 |
| Sulfate adenylyltransferase | EHI_197160 |
| Ras family GTPase | EHI_198330 |
| Polyadenylate-binding protein | EHI_198750 |
| PRA1 family protein | EHI_199660 |
| Signal recognition particle receptor alpha subunit | EHI_200840 |
| ** Okada et al., 2005; Watanabe et al., 2023. | |

| Table S4. Proteins associated with secretion ***. | |
| --- | --- |
| Protein | **Access number** |
| TNase-like domain-containing protein | EHI_005150 |
| 40S ribosomal protein S4 | EHI_008210 |
| Tyrosine-tRNA ligase | EHI_009240 |
| Pyruvate phosphate dikinase | EHI_009530 |
| Tubulin alpha chain | EHI_010530 |
| Elongation factor 1-alpha | EHI_011210 |
| Proteasome endopeptidase complex | EHI_011870 |
| Ribosomal protein S14 | EHI_012360 |
| 60S Ribosomal protein L10a-2 | EHI_012480 |
| 40S Ribosomal protein S6 | EHI_013890 |
| NAD(P) transhydrogenase subunit alpha | EHI_014030 |
| 40S Ribosomal protein S2 | EHI_020280 |
| Calmodulin | EHI_023500 |
| 60S Ribosomal protein L12 | EHI_030710 |
| PPi-type phosphoenolpyruvate carboxykinase 2 | EHI_030750 |
| GTP-binding protein | EHI_031410 |
| Cysteine proteinase 2 | EHI_033710 |
| Ribosomal protein L18a | EHI_035600 |
| V-type ATPase, A subunit | EHI_043010 |
| Malic enzyme | EHI_044970 |
| Actin-related protein 2/3 complex subunit | EHI_045000 |
| Uncharacterized protein | EHI_047800 |
| Lysine-tRNA ligase | EHI_047810 |
| Guanine nucleotide-binding protein subunit beta 2-like 1 | EHI_050550 |
| Pyruvate:ferredoxin oxidoreductase | EHI_051060 |
| Elongation factor 1-alpha | EHI_052400 |
| Heat shock protein 70 | EHI_052860 |
| RAP1 | EHI_058090 |
| Galactose-inhibitable lectin | EHI_058330 |
| EF-hand calcium-binding domain containing protein | EHI_060740 |
| EhRab2C | EHI_067850 |
| 60S Ribosomal protein L5 | EHI_068660 |
| MRH domain-containing protein | EHI_069560 |
| Long-chain-fatty-acid-CoA ligase | EHI_079300 |
| Filopodin | EHI_080740 |
| Small ribosomal subunit protein uS2 | EHI_081410 |
| Coronin | EHI_083590 |
| 4-alpha-glucanotransferase | EHI_084680 |
| Serine-tRNA ligase | EHI_092640 |
| DEAD/DEAH box helicase | EHI_093900 |
| Glycogen phosphorylase | EHI_096830 |
| Glycogen debranching enzyme | EHI_098360 |
| Fructose-1,6-bisphosphate aldolase | EHI_098570 |
| NAD-dependent epimerase/dehydratase domain-containing protein | EHI_098800 |
| NAD(FAD)-dependent dehydrogenase | EHI_099700 |
| Nuclease domain containing protein | EHI_101240 |
| 60S Ribosomal protein L7a | EHI_103310 |
| Cortexillin | EHI_104560 |
| Filamin 2 | EHI_104630 |
| Rab1A | EHI_108610 |
| Phosphoglucomutase | EHI_110120 |
| Myosin heavy chain | EHI_110180 |
| Actin-like protein | EHI_111050 |
| Histone-glutamine methyltransferase | EHI_118840 |
| Alcohol dehydrogenase | EHI_125950 |
| Asparaginyl-tRNA synthetase | EHI_126920 |
| Ribosomal protein L17 | EHI_127330 |
| RACG | EHI_129750 |
| Enolase | EHI_130700 |
| 40S Ribosomal protein S16 | EHI_131190 |
| Protein disulfide-isomerase | EHI_133970 |
| Adenylyl cyclase-associated protein | EHI_136150 |
| Glycogen phosphorylase | EHI_138380 |
| RabC3 | EHI_143650 |
| 40S ribosomal protein S3 | EHI_148320 |
| Gal/GalNAc lectin light subunit | EHI_148790 |
| Ribosomal protein S24 | EHI_148820 |
| 60S acidic ribosomal protein P0 | EHI_148850 |
| Aldehyde-alcohol dehydrogenase 2 | EHI_150490 |
| 40S Ribosomal protein S9 | EHI_152080 |
| Ethanolamine kinase | EHI_152340 |
| Actin-related protein 2/3 complex subunit 4 | EHI_152660 |
| Uncharacterized protein | EHI_160980 |
| Cysteine protease binding protein family 1 | EHI_164800 |
| AP-3 complex subunit delta | EHI_164810 |
| Malate dehydrogenase | EHI_165350 |
| Alcohol dehydrogenase | EHI_166490 |
| PPi-type phosphoenolpyruvate carboxykinase 1 | EHI_166920 |
| Cysteine proteinase 5 | EHI_168240 |
| Aspartyl-tRNA synthetase | EHI_175050 |
| Profilin 1 | EHI_176140 |
| Cdc48-like protein | EHI_176970 |
| 26s proteasome subunit P45 family protein | EHI_177320 |
| 40S ribosomal protein S25 | EHI_177470 |
| Leucine-rich repeat containing protein | EHI_177990 |
| Acetyl-CoA synthetase | EHI_178960 |
| Actin | EHI_182900 |
| Glyceraldehyde-3-phosphate dehydrogenase | EHI_187020 |
| Phosphoglycerate kinase | EHI_188180 |
| V-type ATPase, B subunit | EHI_189850 |
| 40S Ribosomal protein S10 | EHI_197030 |
| Actophorin | EHI_197480 |
| Alcohol dehydrogenase 3 | EHI_198760 |
| Actin-related protein 3 | EHI_198930 |
| PRA1 family protein | EHI_199660 |
| Clathrin heavy chain | EHI_201510 |
| Clathrin heavy chain | EHI_201710 |
| Clathrin heavy chain | EHI_201940 |
| Vacuolar sorting protein 35 (EhVps35) | EHI_002990 |
| *** Sharma et al., 2020 |  |
